# Assessment of inappropriate use of antibiotics and contributing factors in Awi Administrative Zone, Northwestern Amhara regional State, Ethiopia

**DOI:** 10.1016/j.nmni.2024.101557

**Published:** 2024-12-19

**Authors:** Belsti Atnkut, Atalaye Nigussie, Bekele Gebreamanule, Bulti Kumera, Tess Astatkie

**Affiliations:** a Department of Biology, College of Natural and Computational Science, Injibara University, Injibara, Ethiopia; b Department of Statistics, College of Natural and Computational Science, Injibara University, Injibara, Ethiopia; c Faculty of Agriculture, Dalhousie University, Nova Scotia, Canada

**Keywords:** Antimicrobial resistance, Awi zone, Misuse, Pharmaceutical drugs, Self-medication

## Abstract

**Objectives:**

Antibiotic misuse is regarded as the single most significant factor contributing to resistance. Thus, this study aimed to evaluate the prevalence and risk variables linked to the inappropriate use of antibiotics in urban and rural districts of the Awi administrative zone community.

**Methods:**

A total of 1194 rural and urban families, including individuals of various ages and genders from the study area were selected by a multistage stratified random sampling method for a comparative cross-sectional study conducted between December 2022 and June 2023. SPSS version 26 was used to analyze the gathered data. Descriptive statistics and logistic regression analysis methods were used to identify the variables linked to the incorrect use of antibiotics. The adjusted odds ratio was used to calculate the statistical significance of the correlation at a significance level of 5 %.

**Results:**

The findings revealed that, in urban and rural regions, 57.5 % and 69.5 % of the households used unsafe antibiotic practices. The logistic regression analysis showed a significant relationship between the inappropriate use of antibiotics and the household head's age, marital status, family size, monthly income, occupation, educational attainment, place of residence, knowledge of antibiotics, and practice of using antibiotics.

**Conclusion:**

The study area has inappropriate antibiotic use, with statistically significant differences between urban and rural communities. Extensive educational (knowledge and practice) interventions are required to enhance the appropriate use of antibiotics. To guarantee that antibiotics are dispensed correctly and that the right information is provided regarding how the antibiotic functions and should be used, authorized entities should strengthen their regulatory enforcement at pharmacies.

## 1 Introduction

Antimicrobials, such as antibiotics, antifungals, antivirals, antimalarials, and anthelmintics, can interfere with bacteria' natural selection process. These compounds, which can be found in nature or partially or wholly produced in the industry, are used to either kill or stop the growth of germs that cause infectious diseases [1]. An important step forward in tackling the problems of treating, managing, and avoiding major infections is the development of antibiotics. However, the indiscriminate and widespread use of these medications, together with subpar methods for disease prevention and management, has led to the evolution of resistance in a variety of bacterial strains across multiple nations [1].

One of the biggest threats to global public health today is antimicrobial resistance, or the capacity of microorganisms to withstand the drugs meant to destroy them. After their discovery, antibiotics have revolutionized both human and animal health as essential instruments in the fight against deadly infections. On the other hand, because of the emergence and spread of antibiotic resistance, people are dying from illnesses that are incurable in our day and age [2]. The global public health issue of antibiotic resistance has a significant clinical and financial cost [3]. According to the World Health Organization, antibiotic resistance is on the rise worldwide, and this issue leads to higher rates of morbidity and mortality [4].

Antibiotics have an important role in managing and controlling infectious diseases. Many patients' lives have been saved and overall patient care has improved as a result of their use, both in preventive and curative therapy [5]. However, the rise of bacteria resistant to antibiotics poses a danger to their efficacy. Although antimicrobial resistance (AMR) is not new, there is a rise in the number of resistant organisms, the geographic areas where medication resistance is present, and the types of resistance that exist in individual organisms [6]. Diseases and disease agents that were once thought to be controlled by antibiotics are returning to new groups resistant to these treatments.

Antibiotic resistance arises from changes in bacteria that diminish the effectiveness of medications intended to treat infections. The prevalence of antimicrobial resistance (AMR) has escalated due to the widespread and often inappropriate use of antibiotics. Microorganisms exhibiting resistance do not show inhibition at the minimum inhibitory concentration or the effective full concentration of an agent when administered according to a consistent dosing regimen. Resistance to two or more medications or drug classes is referred to as multiple drug resistance, while Cross-resistance refers to an organism's ability to develop resistance to one antibacterial agent while simultaneously gaining resistance to another antimicrobial previously unexposed [7]. Recent statistics underscore the severity of this issue: in the United States, over 2.8 million antimicrobial-resistant infections occur annually, resulting in more than 35,000 deaths each year [8,9]. Globally, AMR was responsible for 1.27 million deaths in 2019 and contributed to nearly 5 million deaths overall [10]. Projections indicate that by 2050, nearly 2 million people could die each year from drug-resistant infections, with a total of 39 million deaths expected over the next 25 years if current trends continue [11]. These alarming figures highlight the urgent need for improved antibiotic stewardship and public health interventions to combat AMR effectively.

One of the biggest challenges facing the world today is estimating the precise economic effects of diseases brought on by resistant bacteria. The need to evaluate the spread of illnesses associated with antibiotic resistance is central to this problem. The global economic impact of antibiotic resistance is significant. 99,000 people die each year in the United States alone as a result of hospital-acquired infections (HAIs) linked to microorganisms resistant to antibiotics [12]. About 50,000 Americans lost their lives to two common HAIs in 2006 pneumonia and sepsis resulting in an estimated $8 billion in costs to the US economy. Hospital stays of at least 13 days are usually necessary for patients suffering from antibiotic-resistant bacterial infections, adding up to an extra 8 million hospital days every year. Reports have indicated costs of up to $29,000 per patient treated for an antibiotic-resistant bacterial infection. Economic losses of approximately $20 billion have been observed in the US, with annual losses of around $35 billion documented in terms of reduced productivity within healthcare institutions due to antibiotic resistance [12].

To prevent resistance from developing and to maximize their medicinal benefits, antibiotics must be used appropriately. Overuse of antibiotics may lead to the development of extended-spectrum beta-lactamases, which can cause resistance [13]. Antimicrobial resistance's global economic effects are still unclear, mainly because the precise cost impact of resistant bacterial illnesses is not exactly known [12]. Even though sickness has always existed in human civilization, it is likely to have a wide range of significant effects on a growing number of people in the future if effective antibiotics are not available. The growing prevalence of infectious, incurable diseases will have a substantial economic impact as more productive people retire from the labor market prematurely. In addition, the growing weight of tending to the ill will put further strain on the community, the individual's family, and larger healthcare institutions. The national production of many countries will decline relative to their current levels due to the cascading effects of this loss of hard labor and the increased burden on health care services. Additionally, the implications for society and culture will be amplified. Apart from the combined consequences of rising rates of illness, the potential arrival of incurable, deadly, and virulent pandemics might have far-reaching consequences, similar to the 1918 worldwide Spanish flu pandemic that claimed the lives of at least 50 million people [14].

Infections resistant to antibiotics are already commonplace worldwide. One of the largest concerns currently posed by gram-positive bacteria is a global pandemic of resistant *S. aureus* and *Enterococcus* species [15]. Drug resistance in common respiratory diseases, such as *Mycobacterium* TB and *Streptococcus pneumonia*, is spreading globally and has become an epidemic [14].

Antimicrobial resistance is a worldwide issue that is spreading quickly, with differing rates of occurrence in different nations [16]. Resistance is closely correlated with the amount of prescription outpatient medication consumed. On the other hand, the use of over-the-counter drugs for self-medication may constitute actual drug consumption. Other sources of self-medication could be prescription drugs left over from earlier therapy sessions or drugs obtained from friends or relatives. It is not advisable to use pharmaceuticals without medical supervision since using the wrong or unnecessary drugs or at inadequate dosages increases the risk of fostering resistant bacteria and the spread of antimicrobial drug resistance [17].

Antimicrobials are also used in agriculture and livestock farming for a variety of purposes, such as prophylaxis (preventive treatment), metaphylaxis (controlling the treatment of an entire herd in the event of a disease outbreak), therapeutic applications (treating sick animals), and growth promotion [18]. Antimicrobial drug resistance (ADR) can compromise health security, disrupt trade, and negatively impact economies in addition to making it more difficult to manage infectious infections. But it is difficult to imagine a world without antibiotics. Without antibiotics, basic medical procedures including routine surgeries, cancer treatments, and organ transplants would not be possible, which could result in deadly situations. Antibiotics must therefore be preserved for certain therapeutic uses. To keep the globe from entering a post-antibiotic future where common diseases and mild injuries could turn deadly, immediate and concerted action is also essential [19].

An increase in AMR occurs naturally [20]. Nevertheless, some human actions hasten the emergence and spread of ADR. Antimicrobial medication overuse in agriculture, fish, poultry, and animal husbandry, together with improper treatment administration, promotes the establishment and selection of resistant strains. ADR also arises and spreads as a result of insufficient infection prevention and control procedures. Prominent institutions including the World Health Organization (WHO), the World Organization for Animal Health (OIE), and the Food and Agriculture Organization (FAO) of the United Nations have partnered to promote optimal methodologies to impede the genesis and dissemination of antibiotic resistance. To address the growing problem of antimicrobial resistance (AMR), ongoing efforts are being made to encourage the appropriate use of antibiotics in both humans and animals.

Antibiotics are used more frequently than all other pharmaceuticals in Ethiopia and in other developing countries with inadequately developed healthcare systems to address health issues to treat bacterial and non-bacterial infections. Furthermore, obtaining these antibiotics without a prescription from a medical expert is a common occurrence, which is made worse by the widespread practice of empirical prescribing without sensitivity testing [21]. Moreover, there are indications of improper antibiotic use in Ethiopia, which may contribute to the emergence of strains of microorganisms resistant to antibiotics. Nevertheless, the extent of inappropriate antibiotic usage and the contributing causes have not been studied in the area. Thus, this study aims to evaluate the inappropriate use of antibiotics and the factors that influence it in both rural and urban areas of Awi administrative zone in Northwest Ethiopia.

## 2 Materials and methods

### 2.1 Study area and period

The study area is Awi Administrative Zone of the Amhara Regional State, Ethiopia. The Amhara Regional State has 15 zones, including three special zones. The distance between Awi Administrative Zone and the capital cities of Ethiopia and the Amhara Regional State is approximately 447 km and 118 km, respectively [22]. Its estimated land size is 857,886 ha, and it is situated between 700 and 2920 m above sea level in the southwest and northwest sections of Ethiopia. Its population in 2018 is predicted to be approximately 1,264,203, of which 1,057,604 are rural residents and 206,599 are urban residents, based on statistics from the 2015 national census and a conversion factor. There are 50.1 % male residents and 49.9 % female residents in the entire population [23]. The zone has a total of 14 districts including the five administrative towns. A comparative cross-sectional study was conducted between December 2022 and June 2023.

### 2.2 Sample size and sampling method

The double population proportion formula was used to determine the sample size, taking into account two comparison groups (urban and rural residents with a 1:2 urban to rural ratio), a 15 % difference between urban and rural populations (P2 = 35 %), a design effect of 2, a presumed inappropriate use of antibiotics practice among the urban community (P1 = 50 %, as no prior study was available), a Z_α/2_ value of 1.96, 80 % power, and a 15 % non-response rate [24]. A total sample size of n = 1194 residents (n_1_ = 398 urban and n_2_ = 796 rural) was chosen based on these presumptions. Sample sizes were assigned proportionately to each district and kebele.

Households from five urban districts (Tilili, Chagni, Dangila, Ankesha, and Injibara) and five rural districts (Guagusa Shikudad, Guanguwa, Dangla, Ayehu Guagusa, and Banja) were selected using a multistage stratified random selection.

### 2.3 Inclusion and exclusion criteria

The inclusion criteria for the assessment of inappropriate antibiotic use and contributing factors in the study area are residents aged 18 years and older who have used antibiotics within the past 6 months and have either sought healthcare for self-medication or prescription. Healthcare workers involved in antibiotic prescription or management, such as doctors, nurses, and pharmacists, are also included.

The exclusion criteria are individuals under 18 years of age, those who have not used antibiotics recently, individuals with severe cognitive impairments, those unwilling to provide informed consent, and healthcare workers not directly involved in antibiotic prescribing or management. Participants already engaged in other similar studies will also be excluded to avoid data overlap. These criteria ensure a representative sample for assessing antibiotic misuse and its contributing factors while maintaining ethical standards.

### 2.4 Data collection instrument

The study employed a pre-tested close-ended questionnaire to collect data on knowledge, antibiotic consumption practices, and socio-demographic characteristics. The questionnaire was initially created in English and then translated into Amharic, the local language. The questionnaire was reviewed by specialists in the domains of microbiology, public health, and statistics, to determine its content validity. A pilot survey was administered to a small group of participants who were not part of the main study. The pilot survey was conducted to ensure the clarity of the questions for the respondents, the feasibility of the survey logistics, and to make any necessary adjustments to the questions or logistics were made based on the results of the pilot survey. Qualified and trained data collectors obtained informed consent from the survey subjects before collecting the data.

### 2.5 Data analysis and process

After manual completeness checks, the collected data were coded and entered into SPSS version 26.0 for analysis. To describe the data, descriptive statistics were used. The Chi-square (χ^2^) test of independence was used to evaluate disparities in the inappropriate use of antibiotics between urban and rural locations. We used both bivariate and multivariate logistic regression models to find factors that indicate inappropriate usage of antibiotics. To demonstrate the significance of the relationship between each component and the dependent variable, adjusted odds ratios (AOR) values, were provided. Every independent variable was tested for significance at a 5 % level of significance.

### 2.6 Operational definition

Using antibiotics for purposes other than those prescribed, getting antibiotics from sources other than healthcare providers, using leftover medications or antibiotics, not finishing the entire course of antibiotics, and self-medication or medicating family members without a prescription from a medical professional are examples of inappropriate use of antimicrobials and antibiotics.

## 3 Results

### 3.1 Descriptive statistics of socio-demographic characteristics of the participant households

The study included n = 1194 households (398 urban and 796 rural; 100 % response rate). There were 820 (68.7 %) male participants and 374 (31.3 %) female participants overall. In terms of age, the majority of the participants were between 30 and 50 years old (40.5 %) and under 30 years old (34.8 %). In terms of family size, 40.5 % of them had <3 and 30.3 % of them had 3-5 family members. The monthly income of around 47.7 % of the participants was less than 3000 Ethiopian Birr (ETB). Just 32.1 % of the participants completed higher education (Table 1).

**Table 1:** Socio-demographic characteristics of the respondents residing in urban and rural districts of the Awi Administrative Zone (n = 1194).

Variable	Urban n (%)	Rural n (%)	Total n (%)
Household head's gender			
Male	196 (49.2)	624 (78.4)	820 (68.7)
Female	202 (50.8)	172 (21.6	374 (31.3)
Household head's age			
<30	33 (8.3)	382 (48.0)	415 (34.8)
30–50	248 (62.3)	236 (29.6)	484 (40.5)
>50	117 (29.4)	178 (22.4)	295 (24.7)
Marital status			
Single	154 (38.7)	412 (51.8)	566 (47.4)
Married	244 (61.3)	384 (48.2)	628 (52.6)
Family size			
<3	87 (21.9)	397 (49.9)	484 (40.5)
3–5	139 (34.9)	223 (28.0)	362 (30.3)
>5	172 (43.2)	176 (22.1)	348 (29.1)
Monthly income (ETB)			
<3000	95 (23.9)	475 (59.7	570 (47.7)
3001–7000	204 (51.3)	190 (23.9)	394 (33.0)
>7000	99 (24.8)	131 (16.5)	230 (19.3)
Religion			
Orthodox	345 (86.7)	572 (71.9)	917 (76.8)
Muslim	36 (9.0)	117 (14.7)	153 (12.8)
Protestant	17 (4.3)	107 (13.4)	124 (10.4)
Occupation			
Merchant	11 (2.8)	161 (20.2)	172 (14.4)
Governmental employee	381 (95.7)	141 (17.7)	522 (43.7)
Farmer	6 (1.5)	494 (62.1)	500 (41.9)
Educational level			
Can't read and write	13 (3.3)	256 (32.2)	269 (22.5)
Primary school	32 (8.0)	255 (32.0)	287 (24.0)
Secondary and preparatory school	131 (32.9)	124 (15.6)	255 (21.4)
College and above	222 (55.8)	161 (20.2)	383 (32.1)

### 3.2 Knowledge of antibiotics use among participants

Majority of the participants preferred self-medication over medical consultation both in urban (69.8 %) and rural (55.5 %) areas. Additionally, 61.7 % of rural and 59.3 % of urban residents self-medicated with antibiotics to treat any ailment. In the investigated locations, the percentage of inappropriate antibiotic usage was 65.5 % overall; in urban and rural locations, the percentages were 57.5 % and 61.5 %, respectively (Table 2). The χ^2^ test of independence results showed that the knowledge levels in urban and rural areas are not similar. The associations between dwelling area (urban or rural) and 1) Are different antibiotics needed to cure different diseases? 2) Can antibiotics kill bacteria? 3) Can self-medication delay seeking medical advice? 4) Do you know what antibiotics are? 5) Do you keep antibiotics at home for later use? 6) Is self-medication better than medical consultation? 7) Do you get antibiotics for self-medication? 8) Do you think self-medication with antibiotics is good? and 9) Reasons for self-medication are highly significant (Table 2). However, the association between dwelling area and 1) Do you stop taking antibiotics before completing the treatment? and 2) Do you use antibiotics through self-medication to treat any illness? are not significant. These results highlight the need for trainings and government interventions customized for urban and rural residents.

**Table 2:** Knowledge of the respondents on antibiotics and antibiotics use among residents of urban and rural districts of Awi administrative zone, and χ^2^ tests of the association between each knowledge categorical variable and dwelling area p-values.

Variable	Urban n (%)	Rural n (%)	Total n (%)	P-value
Are different antibiotics needed to cure different diseases?				<0.001
Yes	397 (99.7)	474 (59.5)	871 (72.9)	
No	1 (1.3)	322 (40.5)	321 (26.9)	
Can antibiotics kill bacteria?				<0.001
Yes/Correct response	280 (70.4)	644 (80.9)	924 (77.4)	
No/Incorrect response	118 (29.6)	152 (19.1)	270 (22.6)	
Can self-medication delay seeking medical advice?				<0.001
Yes	120 (30.2)	448 (56.3)	568 (47.6)	
No	278 (69.8)	348 (43.7)	626 (52.4)	
Do you know what antibiotics are?				<0.001
Yes	267 (67.1)	413 (51.9)	680 (57.0)	
No	131 (28.4)	383 (48.1)	514 (43.0)	
Do you stop taking antibiotics before completing the treatment?				0.120
Yes	189 (47.5)	416 (52.3)	605 (50.7)	
No	209 (52.5)	380 (47.7)	589 (49.3)	
Do you keep antibiotics at home for later use?				<0.001
Yes	13 (3.3)	173 (21.7)	186 (15.6)	
No	385 (96.7)	623 (78.3)	1008 (84.4)	
Is self-medication better than medical consultation?				<0.001
Yes	278 (69.8)	442 (55.5)	720 (60.3)	
No	120 (30.2)	354 (44.5)	474 (39.7)	
Do you use antibiotics through self-medication to treat any illness?				0.426
Yes	236 (59.3)	491 (61.7)	727 (60.9)	
No	162 (40.7)	305 (38.3)	467 (39.1)	
Do you get antibiotics for self-medication?				<0.001
Pharmacy without prescription	206 (51.8)	361 (45.4)	567 (47.5)	
Family	46 (11.6)	187 (23.5)	233 (19.5)	
Friend	100 (25.1)	184 (23.1)	284 (23.8)	
Leftover (previously prescribed)	46 (11.6)	64 (8.0)	110 (9.2)	
Do you think self-medication with antibiotics is good?				0.001
Yes	137 (34.4)	280 (35.2)	417 (34.9)	
No	248 (62.3)	443 (55.6)	691 (57.8)	
Not sure	13 (3.3)	73 (9.2)	86 (7.2)	
Reasons for self-medication				<0.001
Emergency illness	37 (9.3)	193 (24.2)	230 (19.3)	
Experience	206 (51.8)	286 (35.9)	492 (41.2)	
Health facilities being too far	100 (25.1)	277 (34.8)	377 (31.6)	
No medication in health facility	55 (13.8)	40 (5.0)	95 (8.0)	
Antibiotics usage practice				<0.001
Appropriate	169 (42.5)	243 (30.5)	412 (34.5)	
Inappropriate	229 (57.5)	553 (69.5)	782 (65.5)	

### 3.3 Factors associated with inappropriate use of antibiotics

The household head's gender, age, marital status, family size, monthly income, occupation, educational level, place of residence, and knowledge and usage of antibiotics are the factors linked to the inappropriate use of antibiotics, according to the bivariate logistic regression analysis.

The multivariate logistic regression analysis showed that family size, monthly income, religion, occupation, and antibiotics knowledge and utilization practice have statistically significant association with inappropriate use of antibiotics. Those who had between 3 and 5 family size are 0.32 [AOR: 0.32; p-value = 0.026] times more likely (68 % less likely) to use antibiotics inappropriately than those who had >5 family members (Table 3). Respondents whose monthly income is between 3001 and 7000 ETB are 2.66 [AOR: 2.66; p-value = 0.036] times more likely to use antibiotics inappropriately than those whose monthly income is > 7000 ETB. Respondents whose religion is Orthodox are 0.27 [AOR: 0.27; p-value = 0.033] times more likely (73 % less likely) to use antibiotics inappropriately than those whose religion is Protestant. Respondents whose occupation is merchant and governmental employees are 14.61 [AOR: 14.61; p-value = 0.002] and 7.95 [AOR: 7.95; p-value = 0.012] times more likely to use antibiotics inappropriately than those who are farmers, respectively. Those who did stop taking antibiotics before completing the treatment are 0.17 [AOR: 0.17; p-value = 0.022] times more likely to use antibiotics inappropriately than those who did not stop taking antibiotics before completing the treatment. Respondents who keep antibiotics at home for later use are 0.06 [AOR: 0.06; p-value = 0.002] times more likely to use antibiotics inappropriately than those who did not keep antibiotics at home for later use. Those who had no positive response to self-medication with antibiotics are 3.97 [AOR: 3.97; p-value = 0.034] times more likely to use antibiotics inappropriately than those who had a positive response to self-medication with antibiotics. Respondents whose reason for self-medication is “health facilities are too far” and “have experience” are 7.08 [AOR: 7.08; p-value = 0.034] and 0.34 [AOR: 0.34; p-value = 0.027] times more likely to use antibiotics inappropriately than those whose reason is “no medication in health facility”, respectively (Table 3).

**Table 3:** χ^2^ test of independence between the studied categorical variables and antibiotic using practice results, and logistic regression analysis results of associated factors with inappropriate antibiotic using practice.

Variable/Category	Antibiotic using practice		χ^2^ test p-value	AOR (95 % CI)	P-value
	Appropriate (n = 412)	Inappropriate (n = 782)			
Household head's gender			0.699		
Male	280	540		1.37 (0.70–2.67)	0.363
Female	132	242		1.00^a^	
Household head's age			<0.001		
<30	101	314		3.42 (0.54–21.73)	0.192
30–50	176	308		1.01 (0.37–2.75)	0.993
>50	135	160		1.00	
Marital status			0.003		
Single	220	346		1.99 (0.90–4.41)	0.091
Married	192	436		1.00	
Family size			0.002		
<3	142	342		0.81 (0.31–2.13)	0.668
3–5	149	213		0.32 (0.12–0.90)	0.026^b^
>5	121	277		1.00	
Monthly income			<0.001		
<3000	143	427		1.15 (0.54–2.43)	0.722
3001–7000	166	228		2.66 (1.06–6.65)	0.036^b^
>7000	103	127		1.00	
Religion			0.846		
Orthodox	318	599		0.27 (0.08–0.90)	0.033^b^
Muslim	54	99		0.40 (0.08–1.94)	0.258
Protestant	40	84		1.00	
Occupation			<0.001		
Merchant	42	130		14.61 (2.76–77.27)	0.002^b^
Governmental employee	296	226		7.95 (1.58–39.92)	0.012^b^
Farmer	444	56		1.00	
Educational level			<0.001		
Can't read and write	38	231		1.58 (0.56–4.42)	0.384
Primary school	69	218		0.45 (0.16–1.26)	0.128
Secondary and preparatory school	84	171		0.36 (0.12–1.11)	0.076
College and above	221	162		1.00	
Residence			<0.001		
Urban	169	229		1.04 (0.49–2.19)	0.921
Rural	243	553		1.00	
Can antibiotics kill bacteria?			<0.001		
Yes	282	642		1.50 (0.39–5.69)	0.558
No	130	140		1.00	
Do you think self-medication can delay seeking medical advice?			<0.001		
Yes				0.00 (0.00–0.003)	<0.001^b^
No				1.00	
Do you think different antibiotics are needed to cure different diseases?			0.193		
Yes	291	580		0.04 (0.001–0.02)	<0.001^b^
No	121	202		1.00	
Did you stop taking antibiotics before completing the treatment?			<0.001		
Yes	133	472		0.17 (0.04–0.77)	0.022^b^
No	279	310		1.00	
Do you keep antibiotics at home for later use?			<0.001		
Yes	17	169		0.06 (0.01–0.37)	0.002^b^
No	395	613		1.00	
Do you use antibiotics through self-medication to treat any illness?			<0.001		
Yes	212	515		1.07 (0.44–2.55)	0.888
No	200	267		1.00	
Where do you get antibiotics for self-medication?			<0.001		
Pharmacy without prescription	264	303		1.42 (0.43–4.68)	0.565
Family	42	191		0.40 (0.14–1.16)	0.091
Friend	54	230		0.68 (0.26–1.74)	0.420
Leftover from the previous subscription	52	58		1.00	
Do you think self-medication with antibiotics is good?			<0.001		
Yes	306	111		0.57 (0.24–1.35)	0.199
No	79	612		3.97 (1.34–11.71)	0.013^b^
Not sure	27	59		1.00	
Reasons for self-medication			<0.001		
Emergency illness	100	130		1.65 (0.37–7.40)	0.511
Experience	185	307		7.08 (1.16–43.19)	0.034^b^
Health facilities are too far	65	312		0.34 (0.13–0.88)	0.027^b^
No medication in the health facility	62	33		1.00	

CI: Confidence Interval.

a 1.00: Reference category.

b Significant at 5 % level of significance.

## 4 Discussion

The creation and global spread of resistance mechanisms pose a threat to our ability to tackle prevalent infectious diseases, and the frequency of antibiotic resistance is disturbingly high worldwide. Due to the decreasing efficiency of antibiotics, diseases like blood poisoning, pneumonia, TB, and foodborne illnesses are becoming more difficult, if not impossible, to treat [25]. Antibiotic resistance develops more quickly when antibiotics are used inappropriately, excessively, and insufficiently to prevent and control infections [25]. All social classes can take action to lessen the effects and stop the spread of resistance.

Measures can be implemented across all societal strata to mitigate the impact and curtail the dissemination of resistance [26,27]. The percentage of improper antibiotic use in rural areas (69.5 %) and urban areas (57.5 %) in this study did differ significantly. The increased rate of improper usage in rural regions may be attributed to the lower educational attainment and scarcity of healthcare resources in these areas. These numbers are greater than the 17.2 % stated for the region's main city, Bahir Dar [24]. Sociodemographic factors may also be linked to this discrepancy. There was a statistically significant difference in the prevalence of self-medication between urban (69.8 %) and rural (55.5 %) districts. One explanation for this discrepancy might be the closer proximity of urban dwellers to one another, which facilitates information and idea sharing. The results of this study are consistent with self-medication in other areas of Ethiopia [28] and Vietnam [29].

This study indicated that 65.5 % of antibiotics were used inappropriately overall, with self-medication accounting for 60.9 % of the total. This percentage is significantly higher than that of a previous study conducted in Yirgalem town, Ethiopia, that reported 37.9 % [30]. Similar rates of self-medication were observed in Harar, Ethiopia (65 %) [5], and in China (59.4 %) [31]. However, these rates contrast sharply with findings from Portugal (19 %) [32], the Euro-Mediterranean region (19.1 %) [33], Egypt (23.3 %) [34], South Ethiopia (14.5 %) [35] and Italy [36]. These discrepancies could be caused by the community's differing awareness of antibiotics, the study participants' cultural attitudes, access to healthcare facilities, inequalities in socioeconomic and educational backgrounds, and degrees of community awareness.

In contrast, a study in India found that 76 % of antibiotics were inappropriately prescribed [37]. This discrepancy could be related to the reasons behind self-medication, since in India, people tend to do so because they believe they are competent in using medications. On the other hand, this study found that the most common reasons for self-medication were restricted access to healthcare facilities and cost-effectiveness. These variations may also be attributed to changes in regulations and enforcement between nations and regions.

The high rate of self-medication (60.9 % of participants self-administered antibiotics without a prescription) in this study highlights the need for pharmacy staff to closely monitor this widespread practice and for tougher restrictions to be put in place regarding antibiotic dispensing procedures. Fighting antibiotic resistance, a problem that is becoming more and more widespread requires addressing these problems.

The potential for antimicrobial resistance to develop from stopping a full course of therapy poses a danger to the effectiveness of antibiotics [38]. Studies have indicated that lack of understanding and awareness of antibiotics may be the cause of medication discontinuation [39]. When the study participants' symptoms started to get better, 50.7 % of them stopped taking their medications. This dropout rate is higher than that in China [40] and other regions of Africa [41]. These differences might be caused by the study participants' relatively high level of antibiotic use, their perception that treatment should end when symptoms go away, the type of health issues they are experiencing, their understanding of the use of antibiotics, and the degree to which they have received guidance from medical professionals.

This study found the demographic categories that used antibiotics inappropriately. These categories include occupation, religion, family size, and monthly income. Research participants with three to five family members are 0.32 times more likely than those with five or more family members to use antibiotics inappropriately, and those with 3001–7000 ETB monthly income are 2.66 times more likely than those with >7000 ETB monthly income. These findings may be due to financial strain and a lack of knowledge about pertinent information regarding the use of antibiotics. One of the major predictors of improper antibiotic usage in this study was adhering to the Orthodox religion, which may have something to do with one's incapacity to take antibiotics during fasting seasons.

In this study, occupations like government and merchant workers appeared as strong predictors of antibiotic misuse. This could be because their jobs prevent them from visiting healthcare facilities during working hours, thus they end up using antibiotics on their own [42]. One such element that may have contributed is their ability to pay for antibiotics when they start to exhibit symptoms. Unsuitable antibiotic use was also predicted by factors like distance to medical facilities and prior emergency room experiences. People who are dissatisfied with healthcare services may decide to investigate other possibilities instead of obtaining care at facilities.

In addition, actions including keeping medications at home for later use and ending antibiotic use early (50.7 %) were found to be factors affecting improper antibiotic use. This percentage is in line with research from Taiwan (49.8 %) [42]. This stands in stark contrast to what was reported from Namibia, where only 20 % of respondents said they had stopped their treatment too soon [43]. These activities are linked to increased concerns about antimicrobial resistance and have been reported in several studies [14].

Assessing the inappropriate use of antibiotics in the Awi Administrative Zone of Northwestern Amhara, Ethiopia, provides several critical advantages. First, it enhances public health awareness by identifying patterns of misuse, which can inform targeted educational campaigns aimed at both the public and healthcare providers. This awareness is essential for promoting responsible antibiotic use, ultimately reducing the incidence of antimicrobial resistance (AMR) in the community. Additionally, such assessments can help identify specific contributing factors to antibiotic misuse, such as socioeconomic status, access to healthcare, and cultural attitudes toward medication. Understanding these factors allows for the development of tailored interventions that address the root causes of inappropriate use. Furthermore, these assessments can lead to improved regulatory frameworks and policies governing antibiotic dispensing practices, ensuring that antibiotics are used appropriately and effectively while preserving their efficacy for future generations.

Despite its advantages, assessing inappropriate antibiotic use also presents several weaknesses. One significant challenge is the limited access to healthcare resources in rural areas, which can tilt data and hinder effective intervention strategies. The reliance on self-reported data may lead to underreporting or misreporting of antibiotic use behaviors due to social desirability bias or lack of awareness among participants. Additionally, there may be cultural barriers that affect individual's willingness to disclose their antibiotic use practices or seek help from healthcare professionals. The findings may also be influenced by variability in educational levels within the population, leading to inconsistencies in understanding proper antibiotic usage. Finally, addressing the identified issues requires coordinated efforts among various stakeholders, including healthcare providers and policymakers; however, a lack of collaboration can impede progress toward effective solutions for mitigating antibiotic misuse and resistance.

## 5 Conclusion

There is a statistically significant difference in the inappropriate use of antibiotics between urban and rural communities in the study area. The variables with a statistically significant correlation with the inappropriate use of antibiotics were family size, monthly income, religion, occupation, knowledge of antibiotics, and the practice of using antibiotics including storing antibiotics at home for later use, not finishing the treatment, and reasons for self-medication.

The study determined the essential elements for which community interventions should be implemented to lower the inappropriate usage of antibiotics. To decrease the inappropriate use of antibiotics, interventions that consider factors like family size, monthly income, religion, occupation, and household knowledge of antibiotics as well as the use of antibiotics themselves such as stockpiling antibiotics at home for later use, stopping treatment early, and self-medication should be the main focus.

We make the following doable suggestions to enhance the improper use of antibiotics in the study area in light of the study's findings: One way to improve the appropriate use of antibiotics is to: (1) implement widespread educational (knowledge and practice) interventions; (2) have relevant health authorities improve the enforcement of pharmacy regulations to ensure that only prescription antibiotics are dispensed; (3) have patients receive appropriate instructions to minimize antibiotic misuse; (4) enforce antibiotic regulations at the zonal, regional, and national levels to reduce over-the-counter sales that lead to antibiotic self-medication; (5) further study and monitoring are required to explore attitudes and practices to achieve better use and control of antibiotics; and (6) develop and enforce strict regulations that prohibit the use of antibiotics for growth promotion in poultry. This includes requiring veterinary prescriptions for all antibiotic usage and ensuring that antibiotics are only administered for therapeutic purposes and under professional supervision.

## CRediT authorship contribution statement

**Belsti Atnkut:** Writing – original draft, Supervision, Methodology, Formal analysis, Data curation, Conceptualization. **Atalaye Nigussie:** Writing – original draft, Supervision, Methodology, Formal analysis, Data curation, Conceptualization. **Bekele Gebreamanule:** Methodology, Investigation, Formal analysis. **Bulti Kumera:** Writing – review & editing, Supervision, Conceptualization. **Tess Astatkie:** Writing – review & editing, Formal analysis.

## Data availability

The raw data supporting the conclusions of this article will be made available by the corresponding author upon a reasonable request.

## Ethics approval and consent to participate

The researcher obtained permission to conduct the study through a written letter from the Vice Dean of Natural and Computational Science Research, Postgraduate, and Community Service, named Banchalem Kassie. This was a requirement due to the university being newly established in Ethiopia. The letter, written in Amharic with reference number CNCS/PG/R/CS/V/Dean 148/15 and dated January 23, 2023, provided formal authorization to proceed with the research. Verbal consent of the respondents was obtained after briefing them on the study's objectives, and any potential harm their participation might cause them. It was clearly explained to them that they retain the right to decline participation or withdraw from the study, and all information provided by them will be kept confidential.

## Funding

The authors did not receive financial support for this research authorship and publication purpose.

## Declaration of competing interest

The authors declare no conflicts of interest.

## References

[1] Mama M., Mamo A., Usman H., Hussen B., Hussen A., Morka G. (2020). Inappropriate antibiotic use among inpatients attending madda walabu university goba referral hospital, southeast Ethiopia: implication for future use. Infect Drug Resist.

[2] Solomon S., Oliver K. (2014). Antibiotic resistance threats in the United States: stepping back from the brink. Am Fam Physician.

[3] Aldhafar A., Talat W. (2017). Knowledge, attitude, and practice toward the usage of antibiotics among public in al-ahsa, Saudi Arabia. Int J Sci Study.

[4] WHO (2012).

[5] Jifar A., Ayele Y. (2018). Assessment of knowledge, attitude, and practice toward antibiotic use among Harar city and its surrounding community, eastern Ethiopia. Interdiscip Perspect Infect Dis.

[6] Levy S., Marshall B. (2004). Antibacterial resistance worldwide: causes, challenges and responses. Nat Med.

[7] Bisht R., Katiyar A., Singh R., Mittal P. (2009). Antibiotic resistance-a global issue of concern. Asian J Pharm Clin Res.

[8] Ifedinezi O.V., Nnaji N.D., Anumudu C.K., Ekwueme C.T., Uhegwu C.C., Ihenetu F.C. (2024). Environmental antimicrobial resistance: implications for food safety and public health. Antibiotics.

[9] Ahmed S.K., Hussein S., Qurbani K., Ibrahim R.H., Fareeq A., Mahmood K.A. (2024). Antimicrobial resistance: impacts, challenges, and future prospects. J Med Surg Public Health.

[10] Salam M.A., Al-Amin M.Y., Salam M.T., Pawar J.S., Akhter N., Rabaan A.A. (2023). Antimicrobial resistance: a growing serious threat for global public health. Healthcare.

[11] Naghavi M., Vollset S.E., Ikuta K.S., Swetschinski L.R., Gray A.P., Wool E.E. (2024). Global burden of bacterial antimicrobial resistance 1990–2021: a systematic analysis with forecasts to 2050. Lancet.

[12] Aslam B., Wang W., Arshad M., Khurshid M., Muzammil S., Rasool M., Qamar M. (2018). Antibiotic resistance: a rundown of a global crisis. Infect Drug Resist.

[13] Del Arco A., Tortajada B., de la Torre J., Olalla J., Prada J. (2015). The impact of an antimicrobial stewardship programme on the use of antimicrobials and the evolution of drug resistance. Eur J Clin Microbiol Infect Dis.

[14] Jambo A., Edessa D., Adem F., Gashaw T. (2023). Appropriateness of antimicrobial selection for treatment of pneumonia in selected public hospitals of eastern Ethiopia: a cross-sectional study. SAGE Open Med.

[15] CDC CDC looks back at 2013 health challenges, ahead to 2014 health worries. http://www.cdc.gov/media/releases/2013/p1216-eoy2013.html.

[16] Grigoryan L., Haaijer-Ruskamp F., Burgerhof J., Mechtler R., Deschepper R. (2006). Self-medication with antimicrobial drugs in Europe. Emerg Infect Dis.

[17] Usha P., Jose S., Nisha A. (2010). Antimicrobial drug resistance-a global concern. Vet World.

[18] Economou V., Gousia P. (2015). Agriculture and food animals as a source of antimicrobial-resistant bacteria. Infect Drug Resist.

[19] Vadhana P., Singh B., Bharadwaj M., Singh S. (2015). Emergence of herbal antimicrobial drug resistance in clinical bacterial isolates. Pharm Anal Acta.

[20] Fleming A. (1999).

[21] Muhie O.A. (2019). Antibiotic use and resistance pattern in Ethiopia: systematic review and meta-analysis. Int J Microbiol.

[22] Tadesse B.A., Gebreamanule B., Temesgen A.N., Tilahun T., Astatkie T. (2023). Determinants associated with an effective online learning system of a teachers' training college in Awi Zone, Ethiopia during the COVID-19 pandemic. Inter Learn Environ.

[23] Yimenu D.K., Teni F.S., Ebrahim A.J. (2020). Prevalence and predictors of storage of unused medicines among households in northwestern Ethiopia. J Environ Res Public Health.

[24] Gebeyehu E., Bantie L., Azage M. (2015). Inappropriate use of antibiotics and its associated factors among urban and rural communities of Bahir Dar city administration, Northwest Ethiopia. PLoS One.

[25] WHO (2020). Antibiotic resistance. https://www.who.int/news-room/fact-sheets/detail/antibiotic-resistance.

[26] Cizman M. (2003). The use and resistance to antibiotics in the community. Int J Antimicrob Agents.

[27] Nyazema N., Viberg N., Khoza S., Vyas S., Kumaranayake L., Tomson G., Stålsby Lundborg C. (2007). Low sale of antibiotics without prescription: a cross-sectional study in Zimbabwean private pharmacies. J Antimicrob Chemother.

[28] Belachew A.S., Hall L., Selvey A.L. (2022). Community drug retail outlet staff's knowledge, attitudes and practices towards non-prescription antibiotics use and antibiotic resistance in the amhara region, Ethiopia with a focus on non-urban towns. Antimicrob Resist Infect Control.

[29] Okumura J.W., Umenai T. (2002). Drug utilisation and self-medication in rural communities in Vietnam. Soc Sci Med.

[30] Dache A., Dona A., Ejeso A. (2021). Inappropriate use of antibiotics, its reasons and contributing factors among communities of Yirgalem town, Sidama regional state, Ethiopia: a cross-sectional study. SAGE Open Med.

[31] Bi P.T., Parton K.A. (2000). Family self-medication and antibiotics abuse for children and juveniles in a Chinese city. Soc Sci Med.

[32] Ramalhinho I.C., Cavaco A., Cabrita J. (2014). Assessing determinants of self-medication with antibiotics among Portuguese people in the Algarve region. Int J Clin Pharm.

[33] Scicluna E.A., Borg M.A., Gur D., Rasslan O., Taher I., Redjeb S.B., Elnassar Z., Bagatzouni D.P., Daoudet Z. (2009). Self-medication with antibiotics in the ambulatory care setting within the Euro-Mediterranean region; results from the armed project. J Infect Public Health.

[34] Sabry N.A., Dawoud D.M. (2014). Antibiotic dispensing in egyptian community pharmacies: an observational study. Res Social Adm Pharm.

[35] Mossa D.A., Wabe N.T., Angamo M.T. (2012). Self-medication with antibiotics and antimalarials in the community of silte zone, south Ethiopia. TAF Prev Med Bull.

[36] Napolitano F.I., Di Giuseppe G., Angelillo I.F. (2013). Public knowledge, attitudes, and experience regarding the use of antibiotics in Italy. PLoS One.

[37] Chandrakanth P.M., Reddy M.M., Gopinath C. (2016). Assessment of public knowledge and attitude regarding antibiotic use in a tertiary care hospital. Asian J Pharm Clin Res.

[38] Kardas P., Devine S., Golembesky A., Roberts C. (2005). A systematic review and meta-analysis of misuse of antibiotic therapies in the community. Intern J of anti agen.

[39] Yunita S.L., Yang H.W., Chen Y.C., Kao L.T., Lu Y.Z., Wen Y.L., To S.Y., Huang Y.L. (2022). Knowledge and practices related to antibiotic use among women in Malang, Indonesia. Front Pharmacol.

[40] Wun Y.T., Lam K.F., Ho P.L. (2013). The public's perspectives on antibiotic resistance and abuse among Chinese in Hong Kong. Pharmacoepidemiol Drug Saf.

[41] Dale D., Asmamaw G., Etiso T., Bussa Z. (2023). Prevalence of inappropriate drug dose adjustment and associated factors among inpatients with renal impairment in Africa: a systematic review and meta-analysis. SAGE Open Med.

[42] Chen C., Chen Y.M., Hwang K.L. (2005). Behavior, attitudes and knowledge about antibiotic usage among residents of Changhua, Taiwan. J Microbiol Immunol Infect.

[43] Pereko D.D., Lubbe M.S., Essack S.Y. (2015). Public knowledge, attitudes and behaviour towards antibiotic usage in Windhoek, Namibia. S Afr J Infect Dis.

